# ﻿New records of a rare gibberfish, *Gibberichthyslatifrons* (Stephanoberyciformes, Gibberichthyidae), from the South China Sea, with comments on morphological characters

**DOI:** 10.3897/zookeys.1172.102433

**Published:** 2023-07-24

**Authors:** Hsuan-Ching Ho, Yo Su, Nok-Sum Leung, Tzu-Yung Lin

**Affiliations:** 1 Department and Graduate Institute of Aquaculture, National Kaohsiung University of Science Technology, Kaohsiung, Taiwan; 2 National Museum of Marine Biology & Aquarium, Pingtung, Taiwan; 3 Institute of Marine Biology, National Dong Hwa University, Pingtung, Taiwan; 4 Australian Museum, Sydney, Australia; 5 Department of Marine Biotechnology and Resources, National Sun Yat-sen University, Kaohsiung, Taiwan; 6 Department of Oceanography, National Sun Yat-sen University, Kaohsiung, Taiwan

**Keywords:** Biodiversity, biogeography, ichthyology, otolith, Small-scaled gibberfish, taxonomy

## Abstract

The gibberfish *Gibberichthyslatifrons* is recorded for the first time from southwestern Taiwan, northern South China Sea, based on the collection of seven adults and subadults from the Dong-gang fish market, Pingtung. These specimens represent the northernmost extent of the adult range of the species, and they fill a distributional gap in the western Pacific Ocean. Our findings suggest that a population of the species has become recently established in the region. Detailed descriptions, otolith morphology, and fresh coloration are provided with comments on the morphological characteristics of the genus.

## ﻿Introduction

Members of the fish family Gibberichthyidae are known for a long leaf-like pelvic appendage in larvae and juveniles, the so-called kasidoron stage, and the appendage is lost at approximately 21–31 mm standard length (de Sylva and Eschmeyer 1977). The family includes a single genus with two species: *Gibberichthyspumilus* Parr, 1933, widespread in the Atlantic Ocean, and *G.latifrons* (Thorp, 1969), from the Indo-West Pacific Ocean (Kotlyar 2002). *Gibberichthyslatifrons* appears to be rare, with no more than 15 individuals known, all collected from scattered localities associated with islands (de Sylva and Eschmeyer 1977; Kotlyar 2002). Causse (2005) reported the largest known individual (134 mm SL), from French Polynesia. Okiyama et al. (2007) described the “Kasidoron” larvae of *G.latifrons* and suggested that larvae were transported to Japanese waters by the Kuroshio Current, but a local population there has apparently not become established. Since then, no additional information on the species has been recorded, and only two records are known from the northern hemisphere, one from Japan (3 juveniles) (Okiyama et al. 2007) and one from Vietnam (1 adult) (Kotlyar 2002). Previous descriptions of adult *G.latifrons* were brief with only a few diagnostic characters used to separate the two congeners. No complete morphological data set of adults has been provided in earlier publications.

During 2021–2023, we collected seven adults (with developed gonads) and subadults (sex indeterminate) from bycatches of deep bottom trawls that were landed in the Dong-gang fish market in southwestern Taiwan. Detailed descriptions, full morphological data, otolith morphology, and photographs are provided for the first time. These specimens represent a new record for the family, genus, and species in Taiwan. They are also the northernmost collection of adults of the species and the third record in the northern hemisphere. We also discuss the morphological characters of *G.latifrons* and compare them to those of *G.pumilus* based on the previous literature and our new specimens.

## ﻿Material and method

Methods for taking measurements generally follow Kotlyar (1996) and Su et al. (2022). Standard length (**SL**) and head length (**HL**) were used throughout. Rakers on all four gill arches were recorded with only the developed rakers counted; the raker at the angle was included in the count of the lower gill rakers. Measurements were taken with digital calipers rounding to the nearest 0.1 mm. Paired-fin characters are presented as left/right whenever available. Counts of vertebrae and predorsal bones were determined by X-radiograph. Scale pockets were counted when scales were missing from damage by the bottom trawl. Morphometric data are presented as % SL or % HL, unless otherwise indicated. Specimens were deposited in the Pisces Collection of the National Museum of Marine Biology and Aquarium, Pingtung, Taiwan (**NMMB-P**) and the Department of Oceanography, National Sun Yat-sen University, Kaohsiung, Taiwan (**DOS**).

## ﻿Results

### ﻿Family Gibberichthyidae

#### ﻿ Gibberichthys

Taxon classificationAnimaliaStephanoberyciformesGibberichthyidae

Parr, 1933

33160C43-ECB2-531E-AC37-B7E4D45C5779

Gibberichthys Parr, 1933: 5 (type species: Gibberichthyspumilus Parr, 1933; by monotypy); de Sylva and Eschmeyer 1977: 228; Kotlyar 1991: 26; Kotlyar 1996: 256; Okiyama et al. 2007: 45.
Kasidoron Robins & de Sylva, 1965: 190 (type species: Kasidoronedom Robins & de Sylva, 1965 [synonym of G.pumilus], by original designation and monotypy).

##### Diagnosis.

Body moderately long, body depth 3–4 times in SL; head large, its length c. 2.5 in SL, fine spiny ridges on head associated with skull ridges; dorsal and anal fins with anterior spines firmly attached to supporting bones; soft-ray portions of dorsal and anal fins distinctly taller than spinous portions; thin tube-like lateral-line canal with c. 32–36 vertical bars, each bearing 6–8 papillae; scales deciduous, covered by skin; pectoral fin small, its base low in position; pelvic fin small, its origin behind pectoral-fin base; anus situated at mid-point of pelvic fins, well in front of anal-fin origin; caudal fin small, forked; total dorsal-fin elements 14–17, anal-fin elements 11–14; total gill rakers 18–23; pyloric caeca 9–13; total vertebrae 28–32. Coloration dark red to black.

#### ﻿ Gibberichthys latifrons

Taxon classificationAnimaliaStephanoberyciformesGibberichthyidae

(Thorp, 1969)

73FE78CA-2AFF-5F9C-9623-1D6EF36BAC06

**Figure 1.**
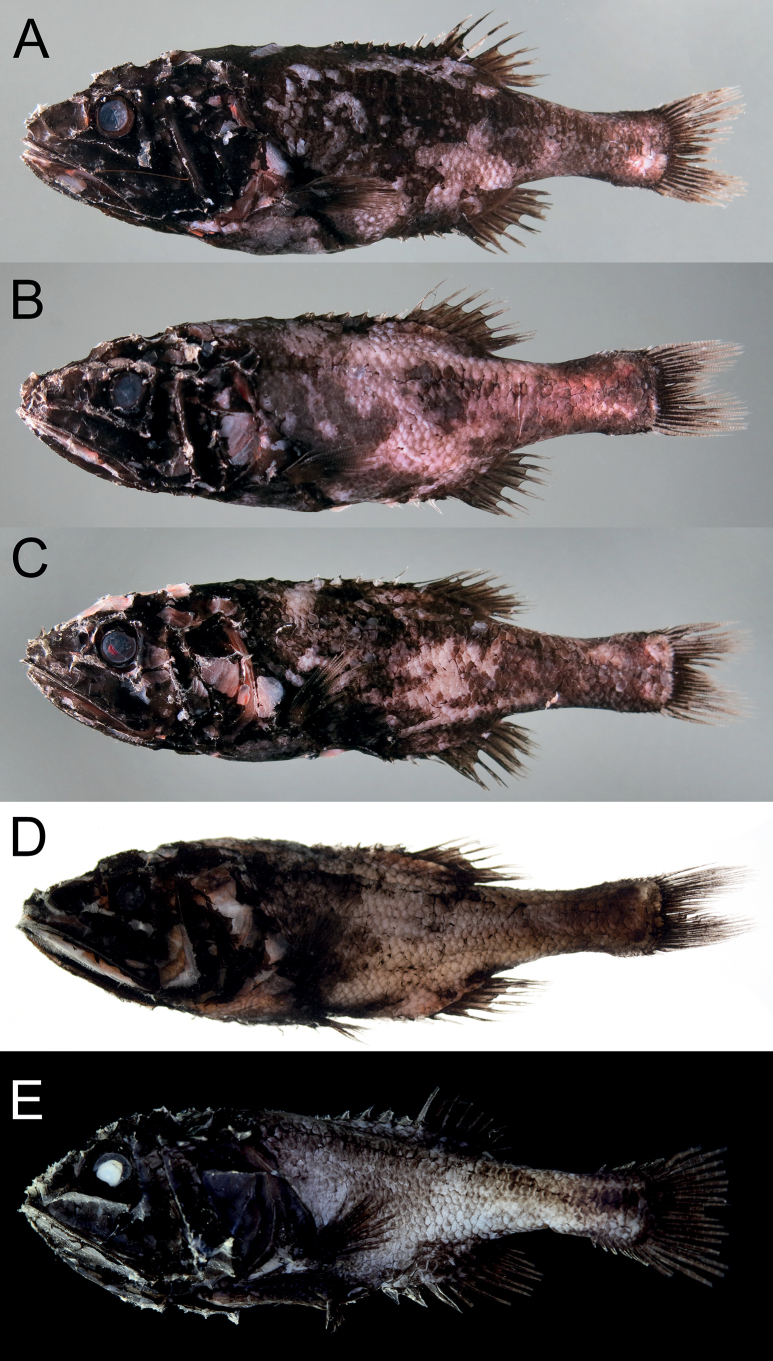
*Gibberichthyslatifrons* (Throp, 1969), fresh condition. **A–C** NMMB-P37470: female, 103.0 mm SL (**A**), male, 101.0 mm SL (**B**), male, 97.2 mm SL (**C**) **D** NMMB-P37435: male, 100.7 mm SL**E**DOS 08997: 49.6 mm SL.

**Figure 2.**
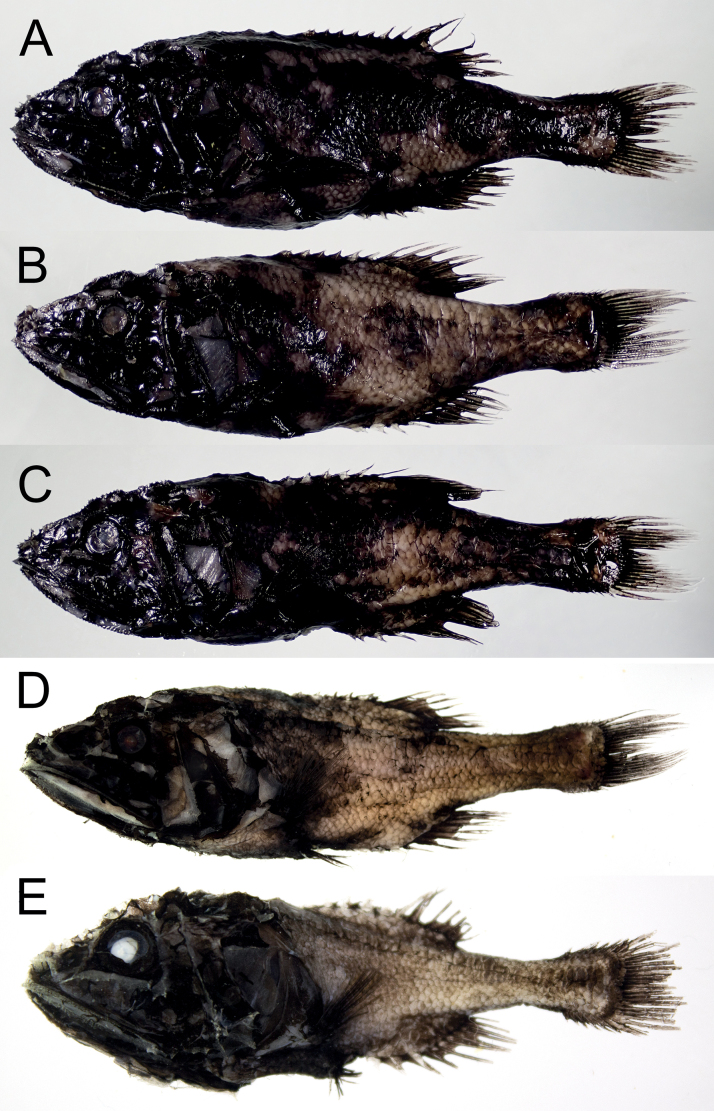
Preserved specimens of *Gibberichthyslatifrons* (Throp, 1969). **A–E** same specimens as in Fig. 1.

**Figure 3.**
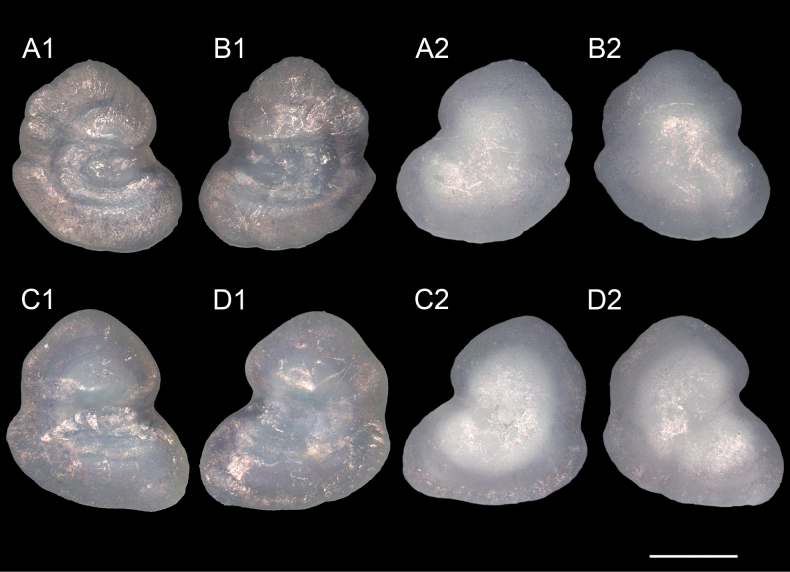
Otoliths of *Gibberichthyslatifrons* (Throp, 1969). **A, B** CHLOL21943, from NMMB-P37470, 101.0 mm SL**C, D** CHLOL22021, from NMMB-P37470, 97.2 mm SL. **A, C** left otolith **B, D** right otolith. **1** inner surface, **2** outer surface. Scale bar: 1 mm.

Figs 1 , 2 , 3 , Tables 1 , 2 English name: Small-scaled gibberfish

Kasidoron latifrons Thorp, 1969: 63, figs 1–4 (type locality: Western Indian Ocean off Zanzibar, Tanzania, 8°34'S, 42°37'E, from stomach content of lancetfish).
Gibberichthys latifrons (Thorp, 1969): de Sylva and Eschmeyer 1977: 228; Kotlyar 1991: 26, 1996: 259, 2002: 478; Causse 2005: 91; Okiyama et al. 2007: 45.

##### Material examined.

DOS 08997 (1, sex indeterminate, 49.6 mm SL), 27 Aug. 2021, coll. N.-S. Leung. DOS 09023 (1, sex indeterminate, 77.6 mm SL), 13 Oct. 2022, c. 400 m, coll. N.-S. Leung. NMMB-P37435 (1 male, 100.7 mm SL), bottom trawl, 26 Dec. 2022, coll. K.-H. Wu. NMMB-P37470 (1 female, 103.0 mm SL and 2 males, 97.2, 101.0 mm SL), bottom trawl, c. 400–500 m, 29 Jan. 2023, coll. H.-C. Ho. NMMB-P37945 (1, sex indeterminate, 82.2 mm SL), 11 Feb. 2023, coll. Y.-M Huang. All from off Dong-gang, Pingtung, southwestern Taiwan. Otoliths: CHLOL21943, taken from NMMB-P37470, 101.0 mm SL, 1.92 mm and 2.03 mm in length. CHLOL22021, taken from NMMB-P37470, 97.4 mm SL, 2.01 mm and 2.12 mm in length.

##### Description of Taiwanese specimens.

Tables 1, 2 provide morphometric and meristic data.

Dorsal-fin elements 5–7 (fixed spines) + 1–2 (movable spines) + 8–9 (soft rays), all rays branched (1 specimen with first ray simple). Pectoral-fin elements 13–14 (1 with 15 in one side), upper and lowermost rays unbranched, others branched. Pelvic-fin elements I, 4–6, spine short and movable, slightly recurved, all rays branched. Anal-fin elements 2–4 (fixed spines) + 1–2 (movable spines) + 7–8 (soft rays). Principal caudal-fin rays 10 + 9, uppermost and lowermost rays unbranched; procurrent caudal-fin rays 7–8 and 6 on upper and lower lobes, respectively. Gill rakers on 1^st^ arch 5–6 + 13–15 = 18–21; on 2^nd^ arch 4–5 + 12–13 = 16–18; on 3^rd^ arch 2 + 10–11 = 12–13; and on 4^th^ arch 0 + 6–8 = 6–8. Lateral line with c. 32–36 vertical branches (some damaged); scale rows along lateral line c. 40–44 (many lost); scale rows above lateral line 5–7; scale rows below lateral line c. 18–21. No abdominal scutes. Pyloric caeca 9–13 (*n* = 4). Vertebrae 12–13 + 18–20 = 30–32 total (*n* = 7); branchiostegal rays 9; interneural 3, upper ends slightly extended upwards on dorsum forming three bumps.

Body moderately long, rather thick, depth at dorsal-fin origin 2.9–3.7 in SL. Head large, length 2.3–2.7 in SL, its height clearly less than its length, 1.3–1.5 in HL; upper profile of head convex, gently curved to dorsal-fin origin; forehead convex and broad, HF1 8.1–10.2 and HF2 4.3–4.6 in HL; eyes small, width 3.7–5.5 in HL; snout convex and short, not extending before premaxilla, length 2.5–3.2 in HL; interorbital space broad, width 2.0–2.5 in HL; postorbital length 1.9–2.1 in HL.

Mouth large, upper-jaw length 1.6–1.7 in HL; posterior end of maxilla rounded, slightly beyond vertical through posterior margin of eye; lower-jaw length slightly larger than upper-jaw length, 1.5–1.6 in HL. Upper jaw overhanging lower jaw completely. Two nostrils at same horizontal level, anterior nostril at about middle of snout; posterior nostril oval, much larger than anterior nostril, right in front of eye. Symphysis of premaxillae forming deep notch, edentate. Symphysis of dentaries with small blunt knob and edentate. Supramaxilla single, with long needle-like process extending anteriorly and long rectangular process posteriorly; covering about third of posterior portion of maxilla in width.

Spiny ridges associated with skull ridge present on head (including snout, jaws, cheek, chin, and gill cover), nape, and pectoral girdle. A small rounded fenestration on each side of supraorbital region, usually covered by skin and can only be observed when skin removed. Outer premaxillary surface completely exposed, extending almost to posterior end of maxilla and bearing villiform teeth along entire length, except for largely naked on middle areas of anterior half, followed by narrow tooth band on posterior half of the bone. Dentary completely covered by upper jaw when mouth closed; a narrow band of villiform teeth extending over about 3/4 of upper jaw. Palatine and vomer toothless.

Gill rakers rod-shaped with pointed tip, somewhat laterally compressed, covered with small teeth on inner surfaces; rakers in outer row of first arch longer than remainder, longest gill raker about equal in length to eye diameter; no raker on inner surface of first arch; gill rakers in outer row of second arch slightly shorter than those in outer row of first arch; gill rakers in outer row of third arch shorter than those in two anterior arches; low knobs present in inner rows of second and third arches; single row of short, somewhat knob-like rakers on fourth arch. Tooth patch present on fifth ceratobranchial. Long, oval tooth patch on third epibranchial arch. Large, triangular villiform tooth patch on second pharyngeal arch and small irregularly triangular tooth patch on third pharyngeal arch. Gill filaments on first arch short, about half length of longest opposite rakers.

Body covered with cycloid scales, all embedded under thin skin. Head entirely naked. Lateral-line scales not distinct from neighboring scales.

Dorsal fin low, with long base, length of dorsal-fin base 3.2–3.7 in SL. Pectoral fin short, its length 2.3–2.5 in HL, tip slightly pointed, reaching to or slightly beyond vertical through anal-fin origin. Pelvic fin short, its length 3.5–4.1 in HL, tip reaching anal-fin origin. Anal fin moderately small, its base length 1.7–2.0 in HL. Caudal fin moderately large, forked, its length 2.2–2.7 in HL.

Lateral line single, originating behind posttemporal bone; its anterior portion slightly curving down, running along dorsal profile on about upper fifth of body, with nearly straight posterior portion ending at caudal-fin base. Clear but weak lateral-line canal along surface of body, with about 32–36 pairs of pale upper and lower vertical extensions, each bearing about 3 papillae (partly damaged by trawl operation in examined specimens).

Anus situated anteriorly, between middle of pelvic fins, clearly anterior to anal-fin origin. Light organ absent. Caudal peduncle long, rather thick, its length 1.4–1.7 in HL, height 3.3–6.1 in HL.

##### Otolith morphology.

Sagittal otoliths were taken from NMMB-P37470, 101.0 mm SL (right: 1.92 mm in length x 2.17 mm in width, left: 2.03 x 2.20) and 97.2 mm SL (right: 2.01 x 2.26, left: 2.12 x 2.28) (Fig. 3). Otolith tall, somewhat bilobed, ventral lobe more developed than dorsal lobe, its width 1.9–2.2% SL, length/depth ratio 0.88–0.93. Dorsal margin highly convex, smooth with 3 or 4 indistinct lobes; ventral margin curved with slight wave; posterior margin entire, slightly convex with a shallow excisura caudalis, pseudo-excisura and pseudo-rostrum poorly developed; distal surface slightly convex, rather smooth; proximal surface slightly concave; sulcus groove deep and wide, with a large ostium opening dorso-anteriorly; ostial colliculum oval, surrounded by broad crista superior and crista inferior; no cauda or caudal colliculum; rostrum broadly rounded; antirostrum rounded, poorly developed; a shallow notch on excisura; dorsal depression deep and broad; ventral depression absent.

##### Coloration.

When fresh, body dark brown to black mixed with deep red color. Mouth cavity and gill chamber orange-red. When preserved, body uniformly black with pale mouth cavity and gill chamber. Stomach and pyloric caeca white, intestine orange-red on anterior half, paler on posterior half. Peritoneal membranes light orange-red on pale gray background. Mouth cavity and gill chamber orange-red on bluish gray background.

##### Maturity.

The largest specimen (103.0 mm SL) is a mature female with two large ovaries containing many large, loose eggs (c. 0.6–0.7 mm). Three other specimens (97.2, 101.0 and 100.7 mm SL) are mature males with well-developed testis.

##### Distribution.

Originally described from the western Indian Ocean (Thorp 1969) and recorded from scattered localities, including Japan, Vietnam, Ceram Sea, Halmahera Sea, New Guinea Sea, off Molucca, Marquesas, Samoa Islands, Comoro Islands, Madagascar, Saya de Malha Bank, and the East Indian Ridge; bathymetric range 320–1700 m (Kotlyar 2002; Causse 2005; Okiyama et al. 2007). Our specimens were collected from depths about 400–500 m by bottom trawl.

## ﻿Discussion

### ﻿Morphometric variations

Table 1 provides the proportional data in *G.latifrons*, including those from the literature. Combining all information, a number of values with growth changes are observed. The head length is relatively long in five smaller specimens (39.3–42.8% SL) and shorter in large individuals (36.5–39.7% SL), the same as the head depth (28.5–33.4%, vs. 23.9–26.4% SL). The eye diameter is largest in the 49.6 mm specimen (11.7% SL), smallest in the 134 mm specimen (5.2% SL), and moderate in other specimens (6.8–8.5% SL). The interorbital width is broadest in the 49.6 mm specimen (21.4% SL), narrowest in the 134 mm specimen (14.7% SL) and moderate in other specimens (15.9–19.0% SL). The lengths of the upper and lower jaws are longest in the 49.6 mm specimen, shortest in the 134 mm SL specimen, and moderate in other specimens. However, the very short lower jaw (18.0% SL) in the 134 mm specimen may be inaccurate, as all other specimens have their lower jaw slightly longer than the upper jaw; four specimens larger than 90 mm SL have their lower-jaw length 24.2–25.0% SL. The very short caudal-peduncle length (15.3% SL) in the 134 mm SL specimen may be also inaccurate; all other specimens measured 24.3–27.1% SL. In general, the smaller specimens tend to have larger proportional measurements which become smaller in adults, reflected by the gradually more-slender body.

**Table 1.** Morphometric data of *Gibberichthyslatifrons*. Abbreviations: A, anal-fin; D, dorsal-fin; PO, pectoral-fin origin; VO, pelvic-fin origin. Data sources: 1. This study, 2. Kotlyar (1985), 3. Kotlyar (2002), 4. Causse (2005).

	*Gibberichthyslatifrons*									
Data sources	1	2	1	1	3	1	1	1	1	4
SL (mm)	49.6	64.0	77.6	82.2	86.0	97.2	100.7	101.0	103.0	134.0
% SL										
HL	42.8	40.6	42.8	39.3	41.9	36.5	39.7	38.7	38.4	38.9
Head depth	33.4	31.3	31.0	31.2	28.5	23.9	26.3	25.5	26.4	24.7
Snout length	13.4	15.6	14.2	14.0	15.7	14.8	14.1	15.2	14.6	12.7
Eye diameter	11.7	7.8	8.3	8.5	7.2	6.8	7.3	7.0	7.1	5.2
Interorbital width	21.4	18.8	19.0	17.6	18.0	16.3	15.9	17	16.5	14.7
Forehead 1	4.6	2.3	4.2	4.6	5.2	3.9	3.7	4.6	4.8	3.5
Forehead 2	9.9	–	9.7	9.4	–	8	8.8	8.9	8.8	–
Postorbital length	22.2	20.3	22.1	19.5	19.2	17.8	19.3	19.2	19.7	20.2
Upper-jaw length	27.1	24.2	25.9	25.1	26.2	22.4	23.5	23.7	22.4	20.4
Lower-jaw length	28.8	26.6	28.2	27.1	27.3	24.2	24.6	25.0	24.5	18.0
Body depth	30.2	29.7	34.8	31.5	30.2	27.1	28.5	28.5	31.7	25.8
Caudal-peduncle length	24.8	26.6	25.9	23.6	25.0	27.0	27.1	23.9	24.3	15.3
Caudal-peduncle depth	7.1	8.6	10.2	7.6	9.3	10.9	8.5	11.4	10.1	9.3
Predorsal length	44.3	46.9	48.1	47.2	49.1	46.0	46.0	44.5	50.1	44.3
Preanal length	57.1	54.7	56.4	60.2	62.3	57.6	56.4	57.6	58.5	48.0
Prepectoral length	46.8	43	44.3	43.4	51.2	41.0	40.1	40.7	41.4	38.9
Prepelvic length	48.5	36.9	48.0	49.7	54.1	47.0	47.2	47.6	50.0	43.2
PO–VO	8.4	4.7	7.5	8.2	7.0	8.8	10.5	8.8	11.5	4.8
Longest D ray	–	–	–	–	–	12.3	14.0	13	11.9	–
Dorsal-fin base	29.6	28.1	30.9	29.4	22.1	29.4	27.2	31	28.2	29.5
Longest A ray	15.0	–	–	–	–	12.8	12.1	12.7	11.7	–
Anal-fin base	23.2	21.9	21.1	22.1	18.6	20.9	20.7	21.2	20.7	22.2
Pectoral-fin length	18.2	21.1	16.3	16.4	19.8	14.7	16.9	16.9	15.9	16.4
Pelvic-fin length	–	10.2	10.8	8.1	11.6	10.0	11.3	9.7	9.3	11.7
Caudal-fin length	–	–	–	–	–	15.9	17.3	17.6	14.4	–

### ﻿Fin elements

There are three interneurals that extend dorsally and form lumps on the midline behind the head in our specimens, but these interneurals are less prominent than those in pre-juveniles observed by de Sylva and Eschmeyer (1977). The author noted that *G.pumilus* has “. . . anterior spines fixed on broad, firm bases; usually, only the last spine is movable. There are usually six spinous points in the dorsal fin and four in the anal fin.” (de Sylva and Eschmeyer 1977: 223). Based on our observation of *G.latifrons*, there are a series of short spines (five to seven on the dorsal fin, two to four on the anal fin) that are firmly attached to their supporting bones (pterygiophores) on the anterior portion of the fins, forming scutes at the fin bases, followed by one or two (on both dorsal and anal fins) longer movable spines; the first two (three in two specimens) spines are small and closely attached, whereas the remainder are well-spaced. As a result, there are a total of seven (eight in one and nine in another specimen) spines on the dorsal fin and four (*n* = 2) or five (*n* = 4) spines on the anal fin. These spines were called “spinous points” in de Sylva and Eschmeyer (1977) but counted as normal spines in Kotlyar (1985, 1996, 2002). These bones supporting the fixed spines are broad and long, identical to the subsequent pterygiophores of the remainder of the fins.

The six larger specimens examined in this study have a normal pelvic fin with the 3^rd^ ray not especially thickened; they do not have a “discontinuity area” as shown by de Sylva and Eschmeyer (1977). The smallest specimen (49.6 mm SL) has a discontinuous area on the first pelvic-fin ray (= second ray in juveniles), which differs from statements in previous publications. It is notable that the fin spine is short and slightly recurved in some specimens.

### ﻿Fenestration on supraorbital region

de Sylva and Eschmeyer (1977) described fenestration on the supraorbital region in juveniles and adults of *G.pumilus*, but this structure was not mentioned in *G.latifrons* by previous authors (Thorp 1969; Kotlyar 1991; Causse 2005; Okiyama et al. 2007). We found one of our specimens with most head skin lost has a fenestration on each side of supraorbital region as shown in *G.pumilus* in de Sylva and Eschmeyer (1977), and further examination reveals that all our specimens have fenestrations which were covered by black skin and cannot be seen without removing the skin. We confirm that fenestration is presented in both species.

### ﻿Meristics

Table 2 provides meristic values of both *Gibberichthys* species, based on our examination and the literature.

**Table 2.** Meristic data of two *Gibberichthys* species. Data source: 1. This study, 2. Kotlyar (1996) 3. Kotlyar (2002), 4. Causse (2005), 5. Okiyama et al. (2007).

	*G.latifrons*						*G.pumilus*
	*n* = 7	*n* = 3	*n* = 1	*n* = 1	Larva (*n* = 1)	Juvenile (*n* = 1)	–
Data sources	1	2	3	4	5	5	2
Dorsal-fin elements	VII–IX, 7–8	V–VIII, 8–9	VIII, 8	VII, 8	12	c. VI, 8	V–VII, 8–9
Pectoral-fin elements	13–14/13–15	12–15	14	–	13/13	14/14	13–15
Pelvic-fin elements	I, 4–6	I, 5–6	I, 5	5	6/6	I, 5/I, 5	I, 5–6
Anal-fin elements	IV–V, 7–8	IV–V, 7–9	V, 7	IV, 7	13	V, 7	IV–V, 7–9
Caudal-fin elements	7–8 + 10 + 9 + 6	–	–	7 + 10 + 9 + 7	7 + 10 + 9 + 5	7 + 10 + 9 + 6	–
Gill rakers on 1^st^ arch	5–6 + 13–15 = 18–21	5–7 + 13–16 = 18–23	6–7 + 14 = 20–21	5 + 13 = 18	21/22	18/17	5–6 + 14–16 = 18–22
Gill rakers on 2^nd^ arch	4–5 + 12–13 = 16–18	–	–	–	–	–	–
Gill rakers on 3^rd^ arch	2 + 10–11 = 12–13	–	–	–	–	–	–
Gill rakers on 4^th^ arch	0 + 6–8 = 6–8	–	–	–	–	–	–
Pseudobranchial filaments	9–11	–	–	–	–	–	–
Lateral-line branches	c. 32–36	33–36	–	–	–	–	28–32
Lateral-line scales	40–44	–	–	–	–	–	–
Scale rows above lateral line	c. 5–7	7–8	–	–	–	–	4
Scale rows below lateral line	c. 15–21	15–16	–	–	–	–	7
Pyloric caeca	9–13	11	–	–	–	–	12–13
Vertebrae	12–13 + 18–20 = 30–32	12 + 20 = 32	14 + 17 = 31	31	–	–	28–29

There are nine to 13 (*n* = 4) pyloric caeca in our specimens, whereas *G.pumilus* has 12 or 13 (de Sylva and Eschmeyer 1977). Kotlyar (1985) counted a total of 23 gill rakers (first arch) in a specimen from the eastern Indian Ocean; Causse (2005) counted 18 in the largest known specimen; Kotlyar (1996) gave a count of 18–23 gill rakers. There are five or six rakers on the upper limb and 13–15 rakers on the lower limb of the first gill arch (total 18–21) in our specimens. de Sylva and Eschmeyer (1977) reported 18–22 gill rakers for both species of *Gibberichthys*.

We counted 14 (*n* = 1), 15 (*n* = 3), or 16 (*n* = 3) total dorsal-fin elements for our specimens. Kotlyar (1985) reported 15 and later (2002) 16; Causse (2005) reported 15. de Sylva and Eschmeyer (1977) reported 13–15 for *G.pumilus* and 15–17 for *G.latifrons*. Although these numbers partly overlap, *G.latifrons* tends to have more total dorsal-fin elements than does *G.pumilus*. We counted 11–13 total anal-fin elements in our specimens, similar to those reported by previous authors (11–13 in *G.pumilus* and 11–14 in *G.latifrons*).

Our specimens have 13–14 pectoral-fin rays (one with 15 in one side), 10 + 9 principal rays, and 7–8 (upper)/6 (lower) procurrent rays on the caudal fin, identical to that reported in the literature. We counted 30 (*n* = 3), 31 (*n*=2) and 32 (*n* = 2) vertebrae in our specimens, which agree with those provided in the literature. de Sylva and Eschmeyer (1977) reported 28–29 total vertebrae for *G.pumilus* and 30–31 for *G.latifrons*. Most previous authors separated the two species by the numbers of vertebrae.

### ﻿Body scales

Although some of the body scales may have been lost due to the trawling operation, many scales remained in our specimens. The scales are cycloid, generally higher than wide, densely overlapping, and covered by thin epidermis all over the entire body, including fin bases. Those associated with the lateral line are about the same size and form as neighboring scales and not especially enlarged; no pored scales were detected. It is notable that Parr (1933) and de Sylva and Eschmeyer (1977) both provided drawings of *G.pumilus* with a hexagonal scale pattern on the body. Whether these hexagonal patterns are formed by scale pockets (i.e., scales lost) or not is uncertain. We did not observe such hexagonal patterns in any of our specimens, possibly because most parts of the bodies were uniformly black except for the scale pockets. The hexagonal pattern was also not mentioned in the literature on *G.latifrons*. In the areas where the scales are lost, the scale pockets formed a somewhat diamond-shaped pattern in our specimens.

### ﻿Lateral line

Based on our examination, there is a thin but clear tube-like canal on the body epidermis that forms the lateral line, plus approximately 32–36 pairs of pale vertical bars, evenly distributed along the upper and lower portion of the lateral-line canal. Each vertical bar bears three black papillae. Kotlyar (1996) reported 33 (33–36 in key) lateral-line scales. However, we counted about 40–44 scales row along the lateral line (beneath the epidermis). It is likely that Kotlyar (1985) counted the vertical bars (or sections) as lateral-line scales. de Sylva and Eschmeyer (1977) counted about 32 vertical rows of six to eight raised papillae on the lateral line but did not provide the number of lateral-line scales. Our specimens have mostly six papillae, three above and three below the lateral-line canal. de Sylva and Eschmeyer (1977) stated that the row of enlarged scales on the lateral line illustrated in Parr (1933) “probably is inaccurate and they believed that two slightly enlarged scales are present…and both scales house papillae of the lateral line.” As mentioned above, the scales along the lateral line do not differ from neighboring scales, and the pores are not associated with the scales beneath them (i.e., no pored scales are present) in our specimens.

### ﻿Head pores

Because our specimens have some damaged skin on the head, it is difficult to observe the exact number of head pores. However, the arrangement of pores is similar to that described in the literature. It is notable that one of our specimens has most of the head skin lost and has white neuromasts along the canal that appear to be associated with head pores.

### ﻿Distribution

Okiyama et al. (2007) suggested that the larvae could be transported northward to Japan by the Kuroshio Current, but only a few young and juveniles have been found in Japanese waters, suggesting a sterile expatriation area for the species. Previous records of adult *G.latifrons* were restricted to tropical areas (17°S–12°N), more often in equatorial regions in the southern hemisphere (Kotlyar 1985, 2002). For nearly two decades of continuous collecting and observing activities in the local fish-landing site in Dong-gang (trawled at c. 22°N), *G.latifrons* specimens were not observed until very recently. Our specimens were collected within a time period of c. 19 months, the second one was collected 14 months after the first, and the last five were collected within three months, thus suggesting that southern Taiwan now has an established adult population of *G.latifrons*. We further suggest that the species has spread northward only recently, possibly because climate change has introduced a warmer hydrographic regime than before, creating a more suitable environment for the species.

## ﻿Conclusions

By consolidating information from the literature with data from our newly collected specimens, we can now confidently distinguish adults and subadults of *G.latifrons* from its only congener *G.pumilus* by the following suite of characters: *G.latifrons* has considerably larger body scales (estimated about twice the size of those in *G.pumilus*), slightly more (14–17) total dorsal-fin elements (vs. 13–15), and 30–32 total vertebrae (vs. 28–29). Our findings begin to fill the gaps in the distribution in the western Pacific Ocean and adult morphology of *G.latifrons*. We also document ontogenetic changes in certain morphometric characters. And we suggest a recent northward distributional expansion into the norther hemisphere waters of southern Taiwan.

## Supplementary Material

XML Treatment for Gibberichthys

XML Treatment for Gibberichthys latifrons

## ﻿Additional information

### Author ORCIDs

Hsuan-Ching Ho https://orcid.org/0000-0003-1154-601X

Yo Su https://orcid.org/0000-0002-3576-9229

Nok-Sum Leung https://orcid.org/0009-0007-3313-3263

Tzu-Yung Lin https://orcid.org/0000-0002-9034-4397

### Data availability

All of the data that support the findings of this study are available in the main text.
